# Supplementary material for: Organic multicomponent microparticle libraries
Source: Nat Commun. 2021 Mar 23;12:1838. doi: 10.1038/s41467-021-22060-z (PMC7988115; doi:10.1038/s41467-021-22060-z)
Supplement: Supplementary file 1 — Supplementary Information [file 41467_2021_22060_MOESM1_ESM.pdf]

Supplementary Information

Organic multicomponent microparticle libraries

Zhang et al.

Table of Contents

1. **Supplementary Method**
2. **Supplementary Figure 1** SEM images of crystalline assemblies of four organic semiconductors involving **1**, **2**, **3**, and **4**.
3. **Supplementary Figure 2** TEM images of crystalline assemblies of four organic semiconductors involving **1**, **2**, **3**, and **4**.
4. **Supplementary Figure 3** PXRD patterns of crystalline assemblies of four organic semiconductors involving **1**, **2**, **3**, and **4** and the diffraction patterns simulated from the single crystal data.
5. **Supplementary Table 1** Summarized crystallographic parameters of four organic semiconductors involving **1**, **2**, **3**, and **4**.
6. **Supplementary Figure 4** Molecular packing motifs and herringbone angles of four organic single crystals involving **1**, **2**, **3**, and **4** based on their crystallographic data.
7. **Supplementary Figure 5** The growth morphologies of **1**, **2**, **3**, and **4** simulated from their crystallographic data using the Material Studio Package.
8. **Supplementary Figure 6** Absorption and PL spectra of the monomer solutions of **1**, **2**, **3**, and **4** in THF as well as PL spectra of their crystalline assemblies.
9. **Supplementary Table 2** Summarized photophysical parameters of four monomer solutions in THF involving **1**, **2**, **3**, and **4** and their crystalline assemblies.
10. **Supplementary Figure 7** SEM images of **34** alloy assemblies with a variable molar ratio of **3**/**4**.
11. **Supplementary Figure 8** Fluorescence microscopy images of **34** alloy assemblies with a variable molar ratio of **3**/**4** and the corresponding PL spectra.
12. **Supplementary Figure 9** PXRD patterns of **34** alloy assemblies with a variable molar ratio of **3**/**4**.

13. **Supplementary Figure 10** SEM images of binary microparticles involving **12**, **1-3**, **1-4**, **34**, **2-3**, and **2-4**.
14. **Supplementary Figure 11** Fluorescence microscopy images, PL spectra, and PXRD patterns of **12** alloy assemblies with a variable molar ratio of **1**/**2**.
15. **Supplementary Figure 12** PL spectra of binary microparticles involving **12**, **1-3**, **1-4**, **34**, **2-3**, **2-4**.
16. **Supplementary Figure 13** PXRD patterns of binary microparticles involving **12**, **1-3**, **1-4**, **34**, **2-3**, **2-4**.
17. **Supplementary Figure 14** Micro-area PL spectra of single **1-3** nanorod heterostructure at different locations.
18. **Supplementary Figure 15** Absorption spectra of the monomer solutions of **3**, **4**, **1**, and **2** in THF as well as PL spectra of their crystalline assemblies.
19. **Supplementary Figure 16** SEM images of ternary microparticles involving **12-3**, **1-34**, **2-34**, **12-4** and quaternary microparticles comprising **1**, **2**, **3**, and **4**.
20. **Supplementary Figure 17** SEM images, PL spectra, and PXRD patterns of **1-34** nanorod heterostructures with a variable molar ratio of **3**/**4** using **1** ribbons as seeds.
21. **Supplementary Figure 18** Fluorescence microscopy images of single typical **12-3** nanorod heterostructures with a variable molar ratio of **1**/**2** using **12** ribbons as seeds and the corresponding PL spectra and PXRD patterns.
22. **Supplementary Figure 19** SEM and fluorescence microscopy images of single typical quaternary microparticles comprising **1**, **2**, **3**, and **4** at y_2 = 10% and x_4 = 50%.
23. **Supplementary Figure 20** Fluorescence and bright-field microscopy images of multiple microparticles involving **1**, **2**, **3**, **4**, **12**, **1-3**, **1-4**, **2-3**, **2-4**, **34**, **12-3**, **1-34**, **2-34**, and **12-34** over a wide scale range.

24. **Supplementary Figure 21** Fluorescence microscopy images of some representative multicomponent microparticles involving **3**, **1-3**, **34**, **1-34**, **2-34**, **12-34**, **3-34**, **1-3-34**, and **12-3-34** after stored in the dark for half a year.
25. **Supplementary Figure 22** SEM, PXRD patterns, and PL spectra of **3-34**, **1-3-34**, **12-3-34** heterostructures.
26. **Supplementary Figure 23** Schematic demonstration of the experimental setup for the optical waveguiding characterization.
27. **Supplementary Figure 24** Bright-field and PL images of **34** alloy microrods formed at x_4 = 0, 0.001%, 0.5%, and 5% by exciting each rod at different positions as well as the corresponding spatially resolved PL spectra, collected from one tip of each microrod with different propagation distance *d*.
28. **Supplementary Table 3** Summarized optical loss coefficient of binary **34** alloy assemblies at x_4 = 0, 0.001%, 0.5%, and 5%.

Supplementary Method

Synthesis of binary, ternary, and quaternary microparticles incorporating core-shell configuration. These multicomponent microparticles were synthesized by a stepwise seeded-growth method. As a typical example, binary **3**-**34** core-shell heterostructures were obtained by using **3** microrods as seeds and **3**/**4** as the shell component. Specifically, the as-prepared **3** microrods were immersed in a monomer solution of **3**/**4** in $CHCl_3$/ethanol ($C_{\mathbf{3}}$ = 2 mM, $x_{\mathbf{4}}$ = 50%, v/v = 1:1) and then the suspension was slowly dried at room temperature. Similar to the preparation procedure of **3**-**34**, **1**-**3**-**34** and **12**-**3**-**34** heterostructures were also synthesized by applying **1**-**3** and **12**-**3** branched heterostructures as seeds, respectively, and **3**/**4** as the shell component. The seed crystals involving **1**-**3** and **12**-**3** branched heterostructures were prepared following the procedures mentioned-above. In particular, the monomer solution of **3**/**4** in $CHCl_3$/ethanol ($C_{\mathbf{3}}$ = 2 mM, $x_{\mathbf{4}}$ = 50%, v/v = 1:1) was used to construct **34** shell layer of **1**-**3**-**34** and **12**-**3**-**34** heterostructures.

Supplementary Figures:

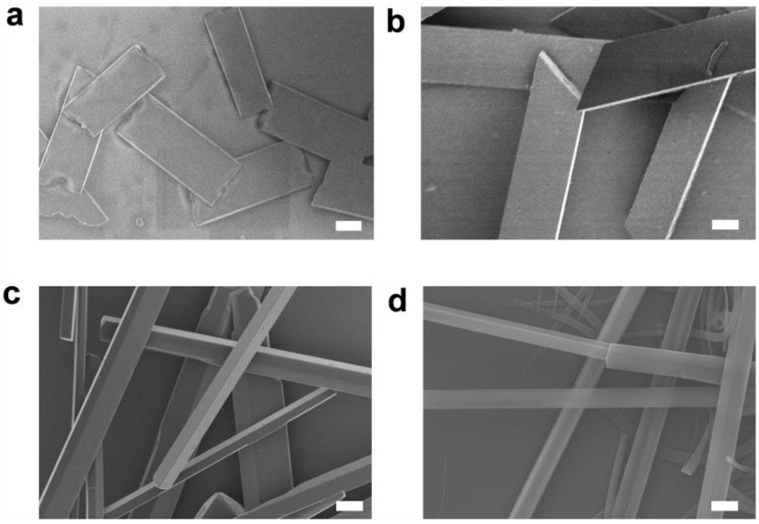

Supplementary Figure 1 SEM images of crystalline assemblies of four organic semiconductors involving (**a**) **1**, (**b**) **2**, (**c**) **3**, and (**d**) **4**. Scale bars, 10 μm.

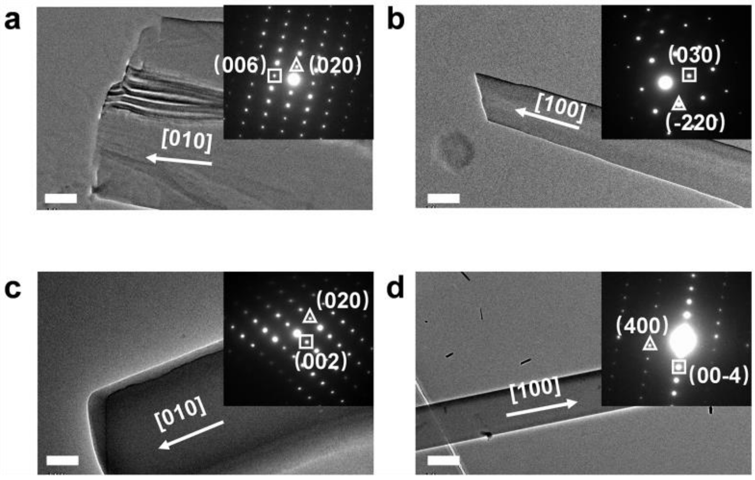

Supplementary Figure 2 TEM images of crystalline assemblies of four organic semiconductors involving (**a**) **1**, (**b**) **2**, (**c**) **3**, and (**d**) **4**. Scale bars, 2 μm. The insets show the corresponding SAED patterns.

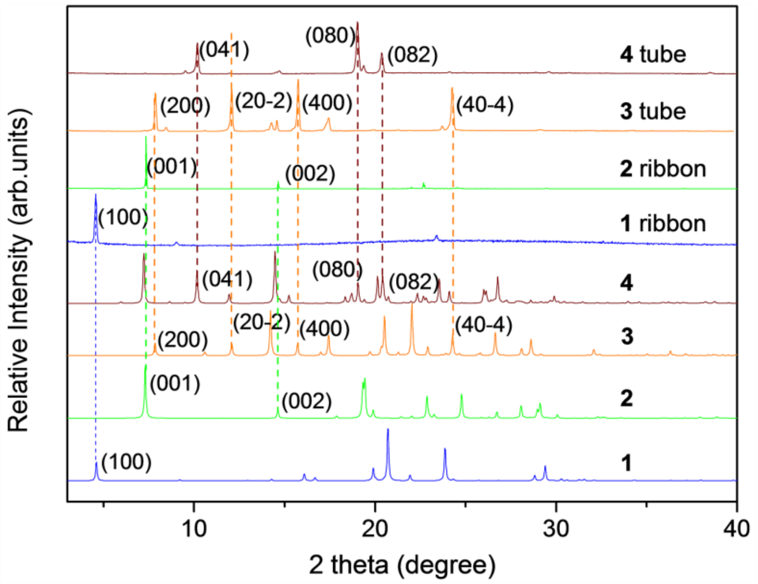

Supplementary Figure 3 PXRD patterns of crystalline assemblies of four organic semiconductors involving **1**, **2**, **3**, and **4** and the diffraction patterns simulated from the single crystal data.

Supplementary Table 1 Summarized crystallographic parameters of four organic semiconductors involving **1**, **2**, **3**, and **4**.

**Molecular crystals**	**1**	**2**	**3**	**4**
Cell Lengths (Å)	a = 19.555(5)	a = 6.0565(9)	a = 22.866(5)	a = 10.126(2)
	b = 7.4506(15)	b = 7.8376(11)	b = 5.3567(11)	b = 37.223(7)
	c = 16.013(4)	c = 13.0104(18)	c = 16.930(3)	c = 24.495(5)
Cell Angles (°)	α = 90	α = 77.127(2)	α = 90	α = 90
	β = 101.04	β = 72.118(2)	β = 99.72(3)	β = 92.93
	γ = 90	γ = 85.792(2)	γ = 90	γ = 90
Cell Volume (Å^3)	2289.86	572.968	2043.92	9220.59

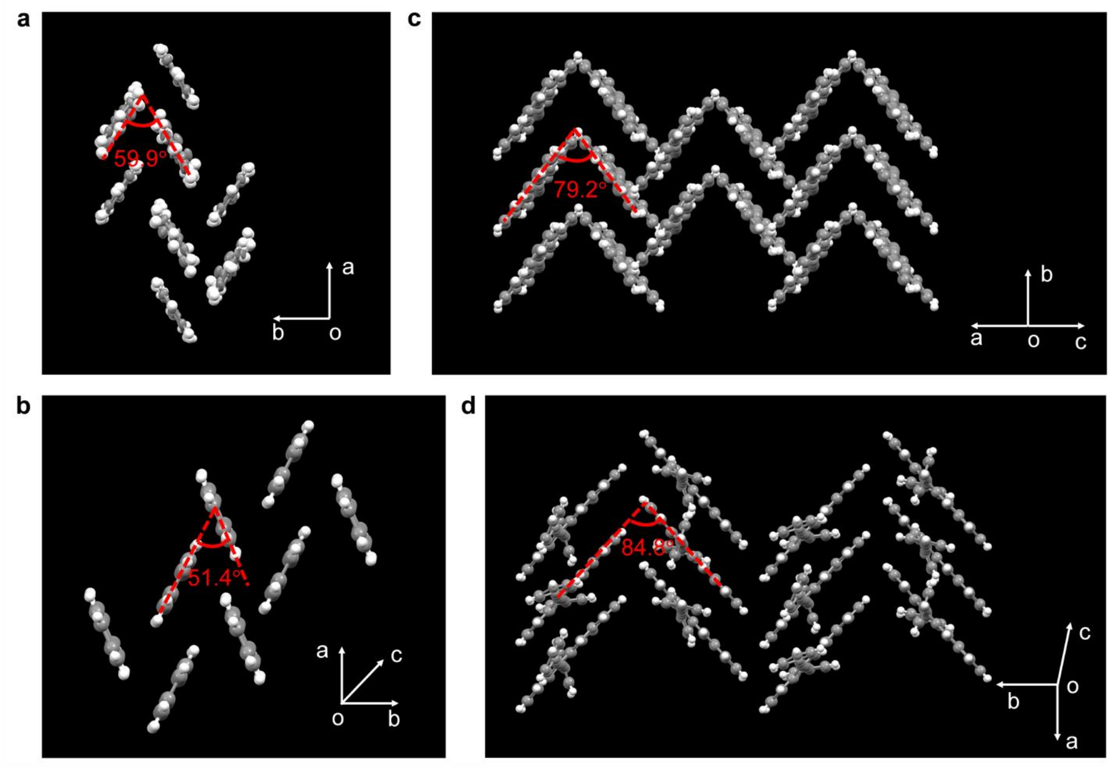

Supplementary Figure 4 Molecular packing motifs and herringbone angles of four organic single crystals involving **1**, **2**, **3**, and **4** based on their crystallographic data.

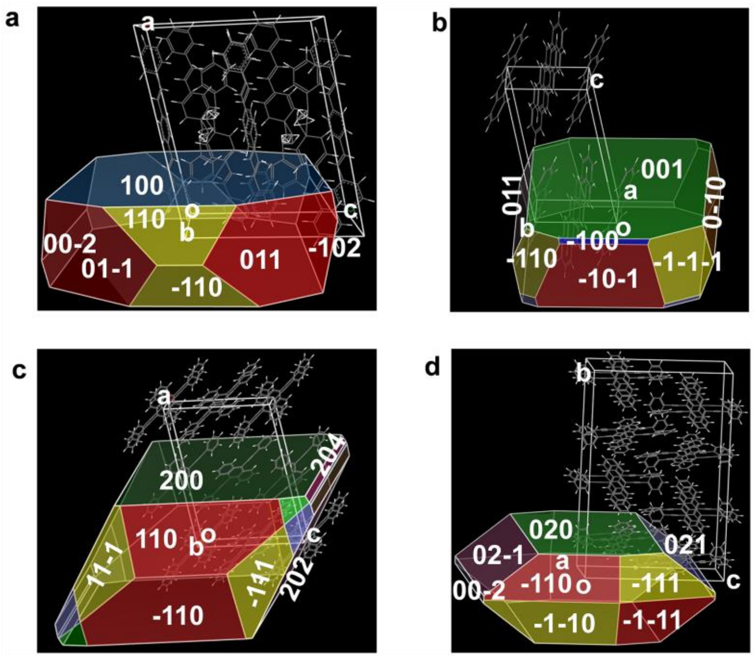

Supplementary Figure 5 The growth morphologies of (**a**) **1**, (**b**) **2**, (**c**) **3**, and (**d**) **4** simulated from their crystallographic data using the Material Studio Package.

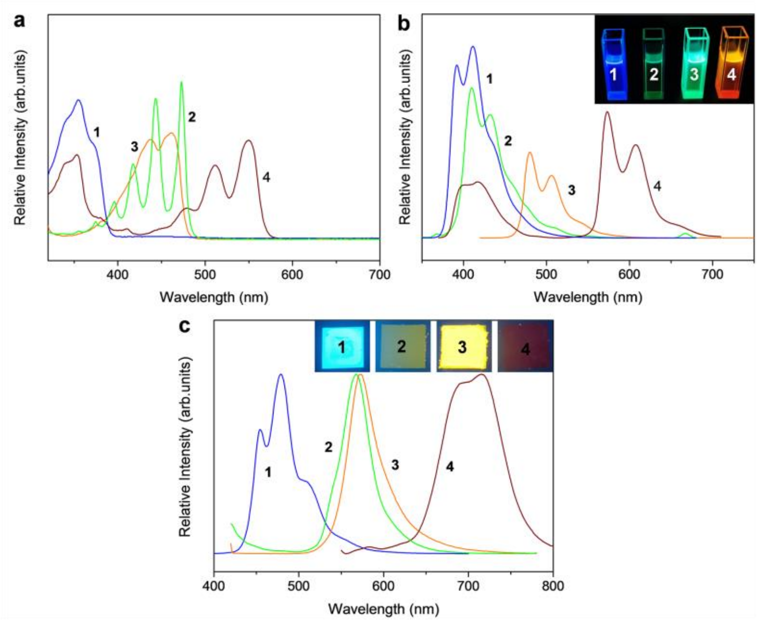

Supplementary Figure 6 (**a**) Absorption and (**b**) PL spectra of the monomer solutions of **1**, **2**, **3**, and **4** in THF as well as (**c**) PL spectra of their crystalline assemblies. The insets shown in **b** and **c** show the photographs of the monomer solutions of **1**, **2**, **3**, and **4** in THF and their crystalline assemblies, respectively, when excited by an UV lamp (365 nm).

Supplementary Table 2 Summarized photophysical parameters of four monomer solutions in THF involving **1**, **2**, **3**, and **4** and their crystalline assemblies.

	**1**	**2**	**3**	**4**
$\lambda_{abs,\ solution}$ (nm)	355, 372	395, 417, 443, 473	439, 461	480, 511, 550
$\lambda_{em,\ solution}$ (nm)	392, 412, 435	410, 432, 510	480, 506	573, 608, 653
$\lambda_{em,\ crystal}$ (nm)	455, 479, 510	568	572	690, 715
PLQYs (%)	78.31	0.41	18.48	2.9

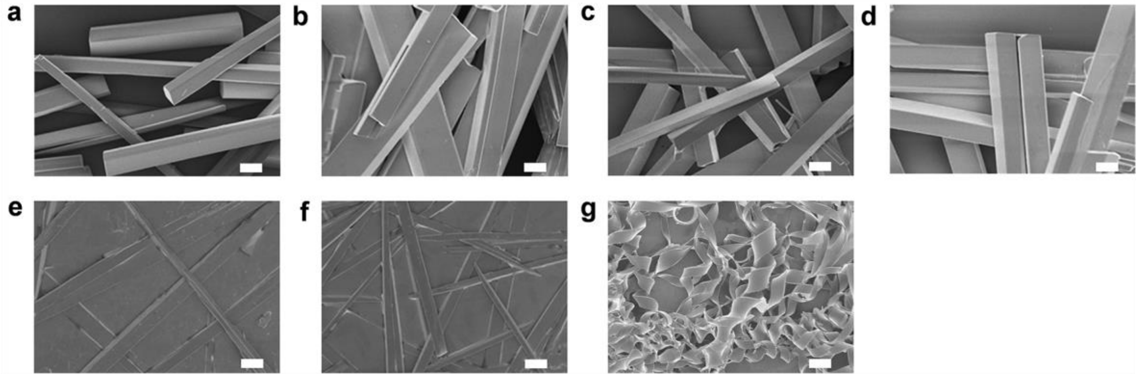

Supplementary Figure 7 SEM images of **34** alloy assemblies with a variable molar ratio of **3**/**4** (x_4 = **a**, 0.0005%, **b**, 0.001%, **c**, 0.01%, **d**, 0.5%, **e**, 5%, **f**, 10%, **g**, 50%). Scale bars, 5 μm.

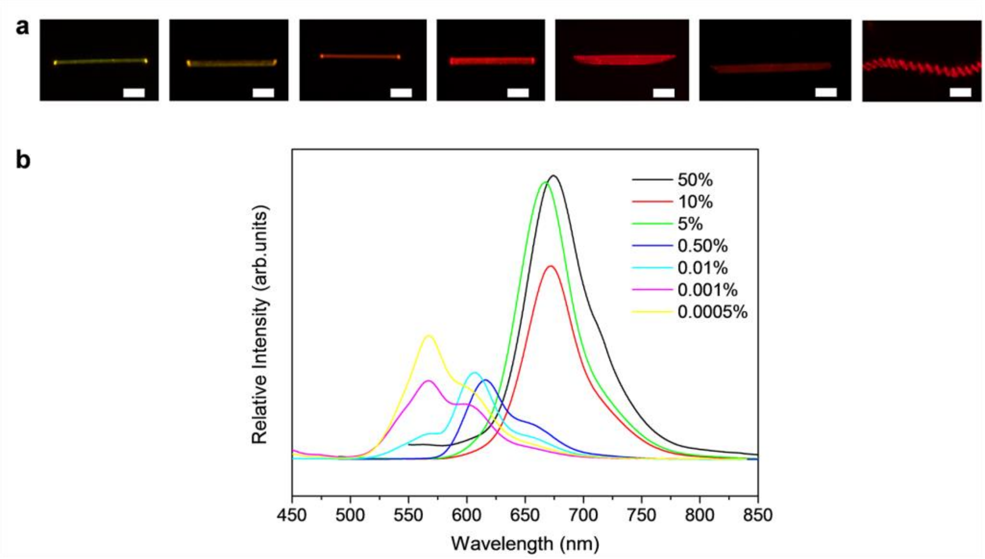

Supplementary Figure 8 (**a**) Fluorescence microscopy images of **34** alloy assemblies with a variable molar ratio of **3**/**4** (x_4 = 0.0005%, 0.001%, 0.01%, 0.5%, 5%, 10%, 50%). The **34** alloy assemblies obtained at 0.0005% ≤ x_4 ≤ 10% were excited with UV light; whereas the alloy helix formed at x_4 = 50% was excited with green light. Scale bars, 5 μm. (**b**) The corresponding PL spectra.

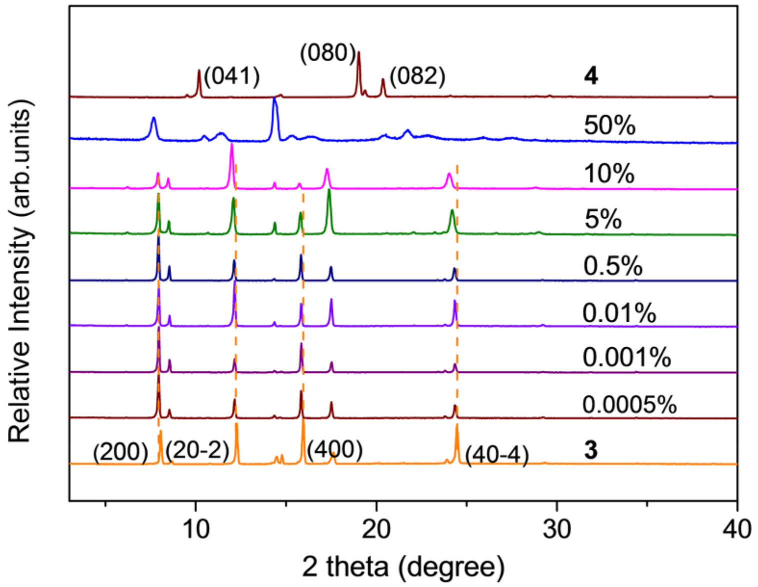

Supplementary Figure 9 PXRD patterns of **34** alloy assemblies with a variable molar ratio of **3**/**4** (x_4 = 0, 0.0005%, 0.001%, 0.01%, 0.5%, 5%, 10%, 50%, 100%).

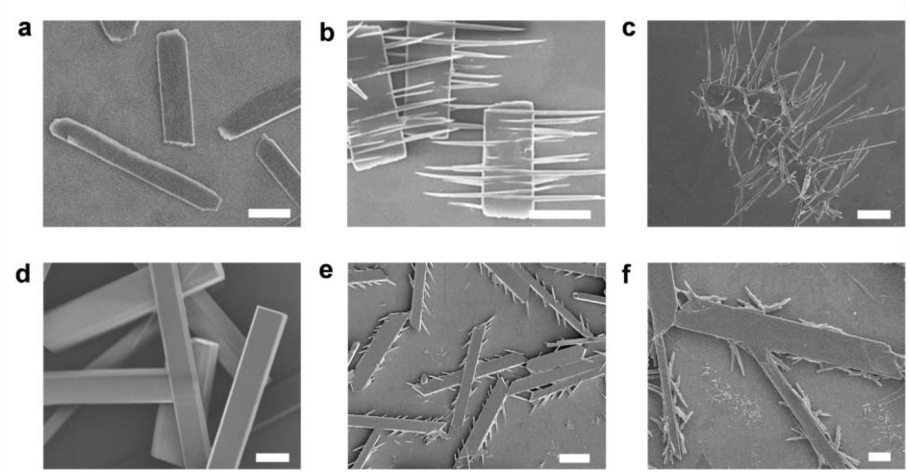

Supplementary Figure 10 SEM images of binary microparticles involving (**a**) **12** (y_2 = 1%), (**b**) **1-3**, (**c**) **1-4**, (**d**) **34** (x_4 = 0.001%), (**e**) **2-3**, (**f**) **2-4**. Scale bars, 10 µm.

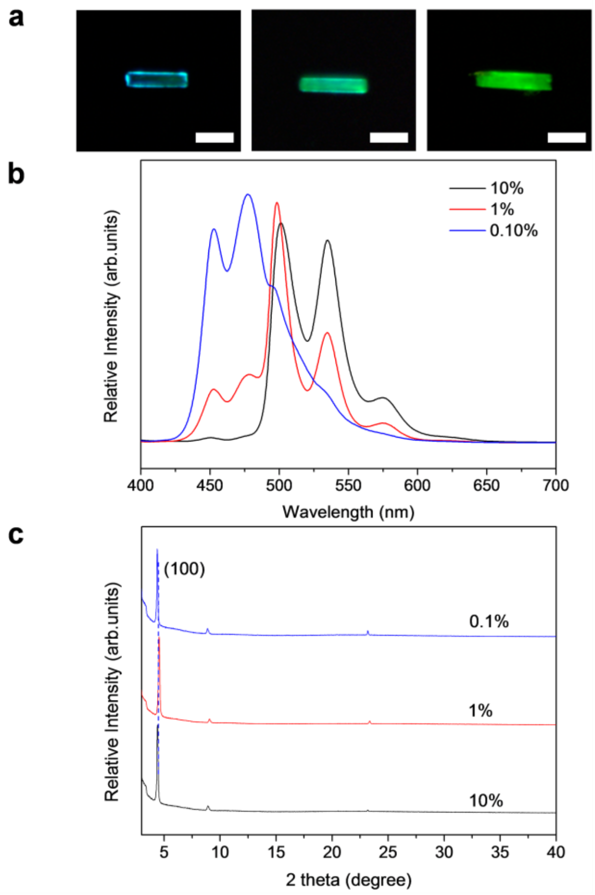

Supplementary Figure 11 (**a**) Fluorescence microscopy images, (**b**) PL spectra, and (**c**) PXRD patterns of **12** alloy assemblies with a variable molar ratio of **1**/**2** (y_2 = 0.1%, 1%, 10%). The samples were excited with UV light. Scale bars, 10 μm.

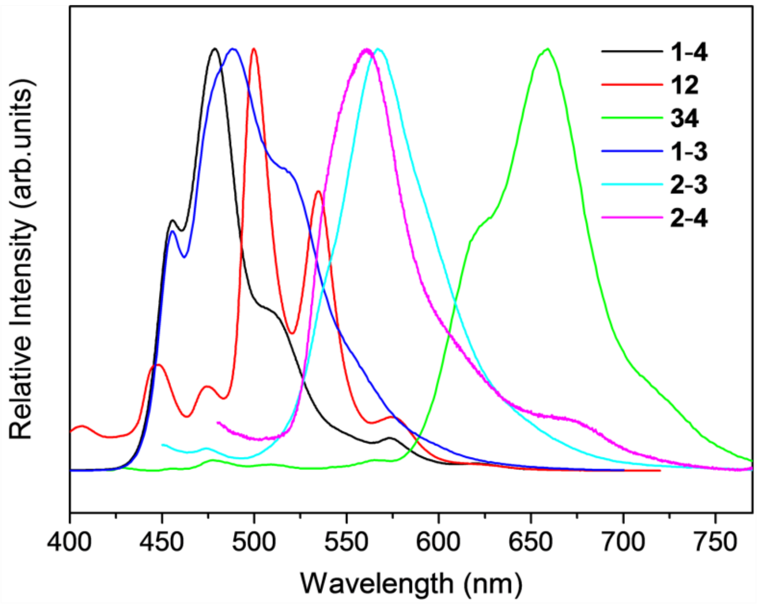

Supplementary Figure 12 PL spectra of binary microparticles involving **12** (y_2 = 1%), **1-3**, **1-4**, **34** (x_4 = 10%), **2-3**, and **2-4**.

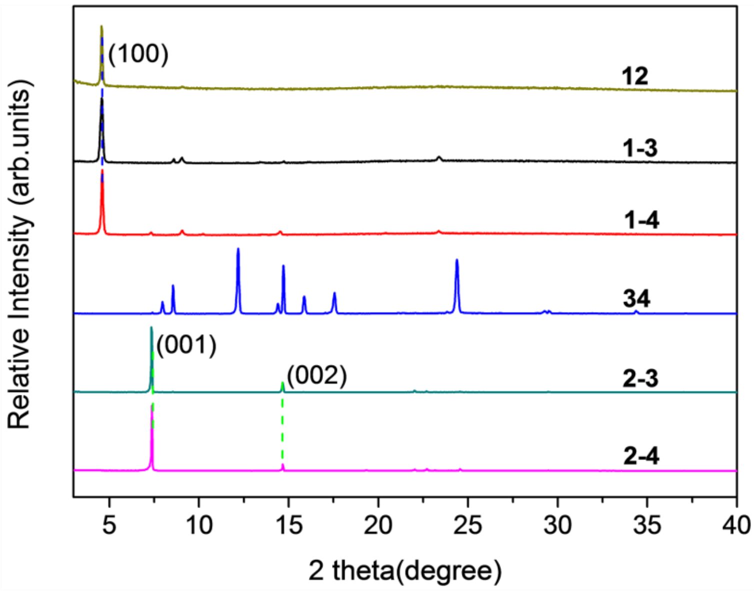

Supplementary Figure 13 PXRD patterns of binary microparticles involving **12** (y_2 = 1%), **1-3**, **1-4**, **34** (x_4 = 5%), **2-3**, and **2-4**.

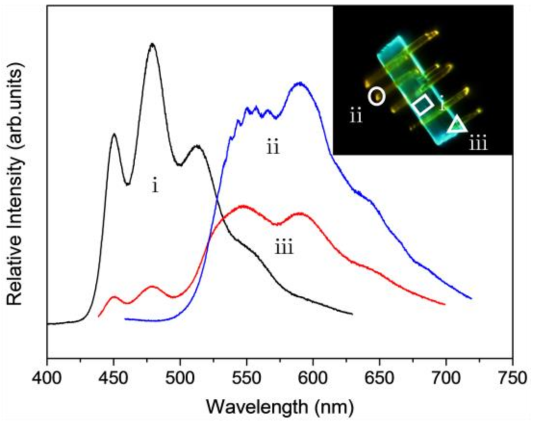

Supplementary Figure 14 Micro-area PL spectra of single **1-3** nanorod heterostructure at different locations. Locations **i** and **ii** represent the main body of **1** ribbon and a rod tip of **3**, respectively. And location **iii** is the junction between the main body of **1** ribbon and a rod tip of **3**. The inset shows the corresponding fluorescence microscopy image.

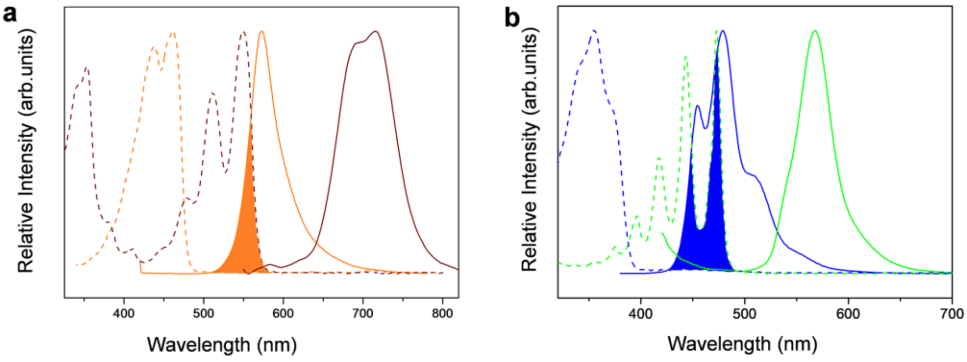

Supplementary Figure 15 Absorption spectra of the monomer solutions of (**a**, orange dashed curve) **3**, (**a**, brown dashed curve) **4**, (**b**, blue dashed curve) **1**, and (**b**, green dashed curve) **2** in THF as well as PL spectra of their crystalline assemblies (**a**, orange solid curve for **3**; **a**, brown solid curve for **4**; **b**, blue solid curve for **1**; **b**, green solid curve for **2**). The shadow regions in (**a**) and (**b**) represent the overlap area of PL spectrum of **3** and absorption spectrum of **4** or that of PL spectrum of **1** and absorption spectrum of **2**.

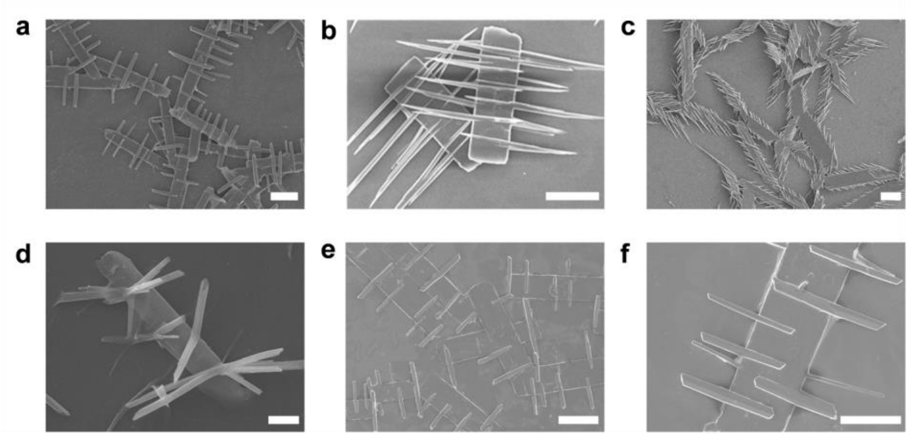

Supplementary Figure 16 SEM images of ternary microparticles involving (**a**) **12-3** (y_2 = 5%), (**b**) **1-34** (x_4 = 5%), (**c**) **2-34** (x_4 = 0.5%), (**d**) **12-4** (y_2 = 5%) and (**e,f**) quaternary microparticles comprising **1**, **2**, **3**, and **4** at (**e**) low- and (**f**) high-magnification. Scale bars, 10 μm.

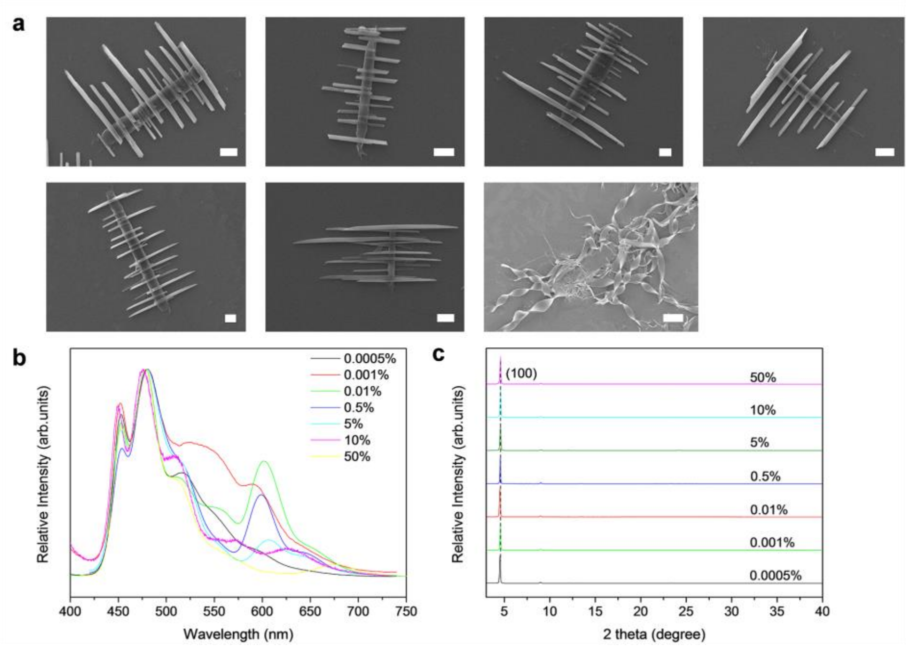

Supplementary Figure 17 (**a**) SEM images, (**b**) PL spectra, and (**c**) PXRD patterns of **1-34** nanorod heterostructures with a variable molar ratio of **3**/**4** (x_4 = 0.0005%, 0.001%, 0.01%, 0.5%, 5%, 10%, 50%) using **1** ribbons as seeds. Scale bars, 10 μm.

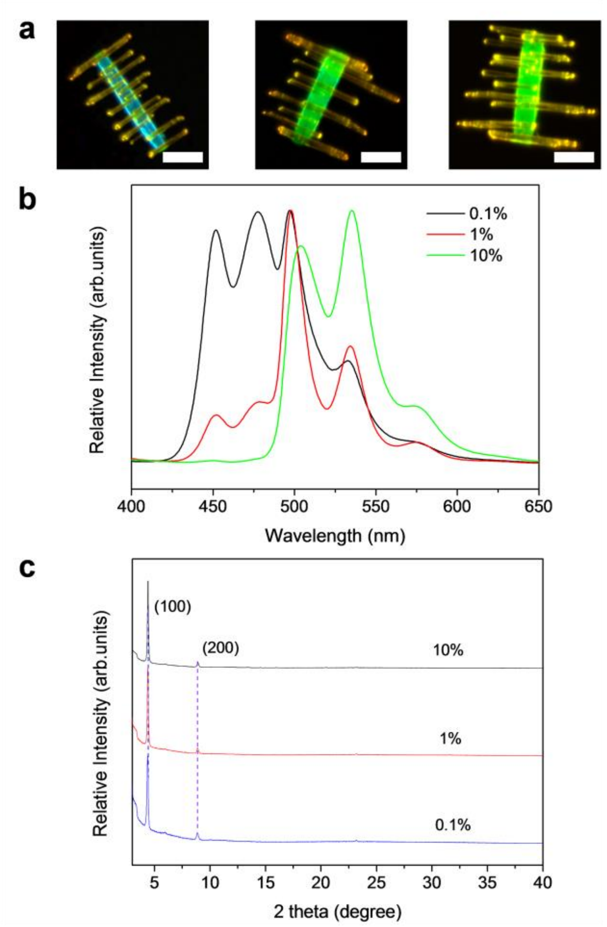

Supplementary Figure 18 (**a**) Fluorescence microscopy images of single typical **12**-**3** nanorod heterostructures with a variable molar ratio of **1**/**2** (y_2 = 0.1%, 1%, 10%) using **12** ribbons as seeds. Scale bars, 10 µm. The corresponding (**b**) PL spectra and (**c**) PXRD patterns.

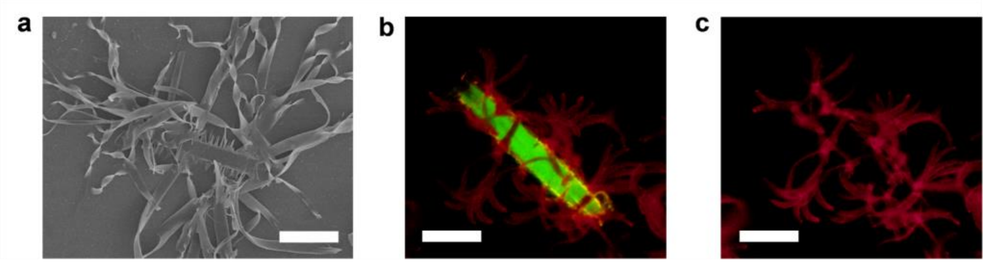

Supplementary Figure 19 (**a**) SEM and (**b**,**c**) fluorescence microscopy images of single typical quaternary microparticles comprising **1**, **2**, **3**, and **4** at y_2 = 10% and x_4 = 50%. The sample was excited with (**b**) blue and (**c**) green light, respectively. Scale bars, 20 μm.

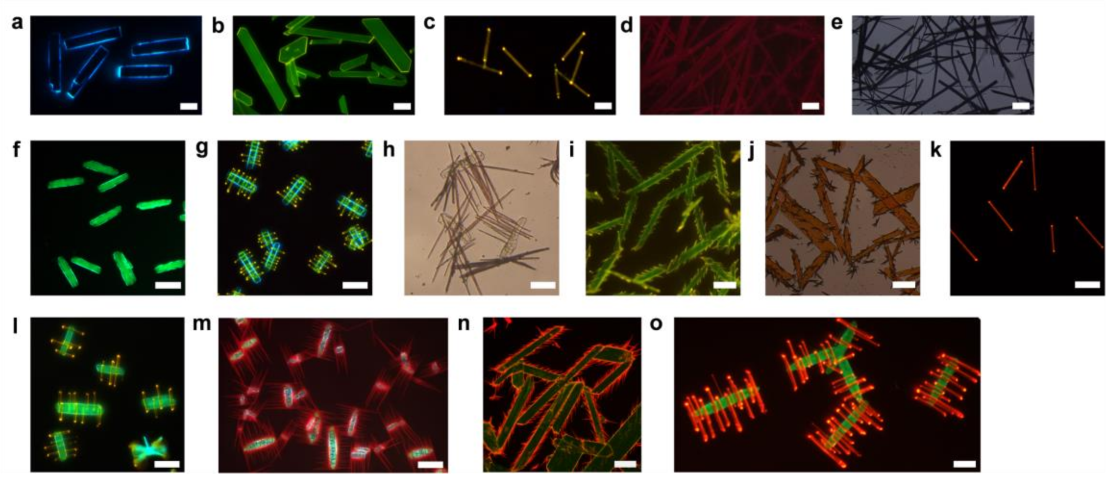

Supplementary Figure 20 (**a**,**b**,**c**,**d**,**f**,**g**,**i**,**k**,**l**,**m**,**n**,**o**) Fluorescence and (**e**,**h**,**j**) bright-field microscopy images of multiple microparticles involving (**a**) **1**, (**b**) **2**, (**c**) **3**, (**d**) **4**, (**e**) **4**, (**f**) **12** (y_2 = 5%), (**g**) **1-3**, (**h**) **1-4**, (**i**) **2-3**, (**j**) **2-4**, (**k**) **34** (x_4 = 0.05%), (**l**) **12-3** (y_2 = 5%), (**m**) **1-34** (x_4 = 10%), (**n**) **2-34** (x_4 = 0.5%), and (**o**) **12-34** (y_2 = 10%, x_4 = 0.5%) over a wide scale range. Scale bars, 10 μm. Among them, the particles **1**, **2**, **3**, **12**, **1-3**, **2-3**, **34**, **12-3**, **1-34**, **2-34** and **12-34** were excited by UV light; while the particle **4** (**d**) was excited by green light.

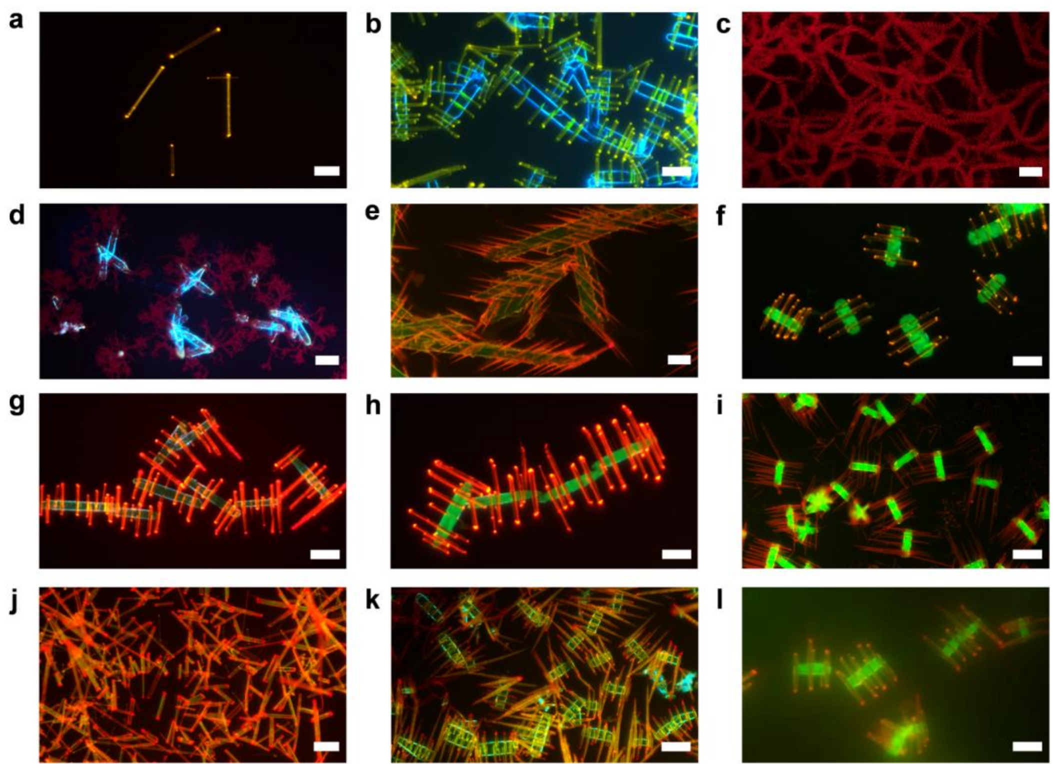

Supplementary Figure 21 Fluorescence microscopy images of some representative multicomponent microparticles involving (**a**) **3**, (**b**) **1-3**, (**c**) **34** (x_4 = 50%), (**d**) **1-34** (x_4 = 50%), (**e**) **2-34** (x_4 = 5%), (**f**) **12-34** (y_2 = 10%, x_4 = 0.001%), (**g**) **12-34** (y_2 = 0.1%, x_4 = 5%), (**h**) **12-34** (y_2 = 5%, x_4 = 5%), (**i**) **12-34** (y_2 = 10%, x_4 = 20%), (**j**) **3-34** (x_4 = 50%), (**k**) **1-3-34** (x_4 = 50%), and (**l**) **12-3-34** (y_2 = 10%, x_4 = 50%) after stored in the dark for half a year. Scale bars, 10 µm.

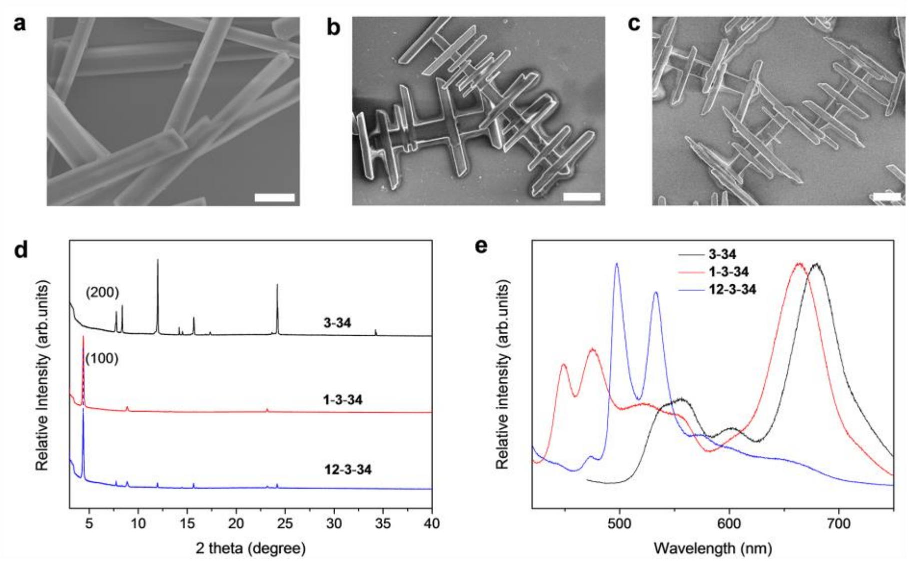

Supplementary Figure 22 (**a-c**) SEM, (**d**) PXRD patterns, and (**e**) PL spectra of (**a**) **3-34** (x_4 = 50%), (**b**) **1-3-34** (x_4 = 50%), (**c**) **12-3-34** (y_2 = 10%, x_4 = 50%) heterostructures. Scale bars, 20 μm.

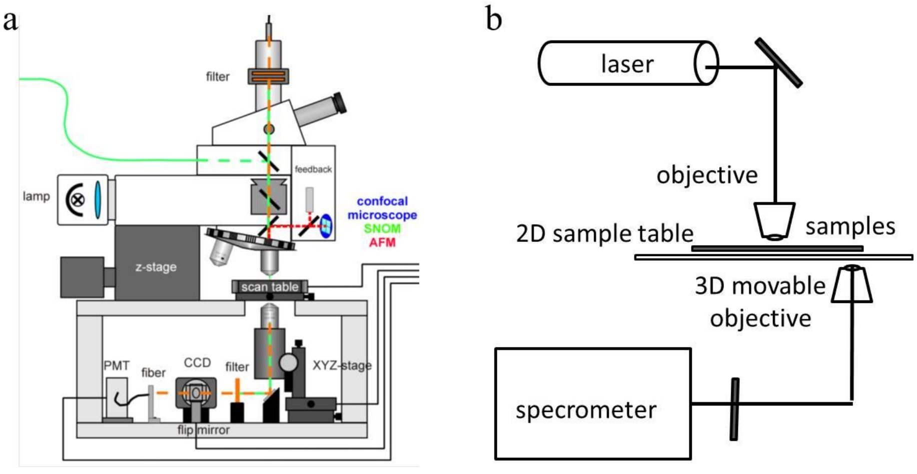

Supplementary Figure 23 Schematic demonstration of the experimental setup for the optical waveguiding characterization: (**a**) the confocal optical microscopy and (**b**) the transmittance optical path for the waveguide measurements.

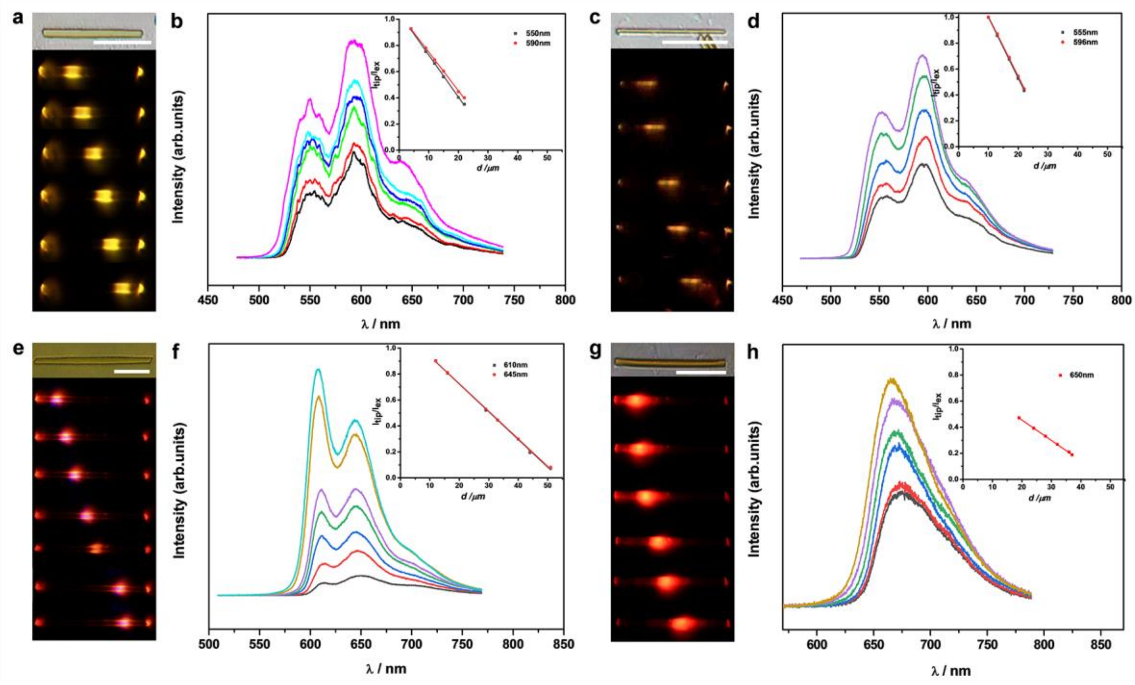

Supplementary Figure 24 Bright-field and PL images of **34** alloy microrods formed at x_4 = (**a**) 0, (**c**) 0.001%, (**e**) 0.5%, and (**g**) 5% by exciting each rod at different positions. Scale bars, 20 μm. (**b**, **d**, **f**, **h**) The corresponding spatially resolved PL spectra, collected from one tip of each microrod with variable propagation distance *d*. The insets display the logarithmic relation of I_{tip}/I_{ex} vs d at different PL peaks, where I_{tip} and I_{ex} are the PL intensity at the output tip and the excited spot, respectively.

Supplementary Table 3 Summarized optical loss coefficient of binary **34** alloy assemblies at x_4 = 0, 0.001%, 0.5%, and 5%.

	**34**			
	x_4 = 0	x_4 = 0.001%	x_4 = 0.5%	x_4 = 5%
λ_{em} (nm)	550, 590	555, 596	610, 645	650
α (dB mm^{-1})	32.0, 29.5	47.1, 46.1	21.3, 21.2	15.6
