# Supplementary material for: Organic multicomponent microparticle libraries
Source: Nat Commun. 2021 Mar 23;12:1838. doi: 10.1038/s41467-021-22060-z (PMC7988115; doi:10.1038/s41467-021-22060-z)
Supplement: Supplementary file 3 — Description of Additional Supplementary Files [file 41467_2021_22060_MOESM3_ESM.pdf]

Description of Additional Supplementary Files

File Name: Supplementary Movie 1

Description: Real-time evolution process of **34** alloy assemblies formed by one-pot method. The sample was excited with UV light.

File Name: Supplementary Movie 2

Description: Real-time evolution process of **1-3** branched nanorod heterostructures formed by one-pot method.

File Name: Supplementary Movie 3

Description: Real-time evolution process of **12** alloy assemblies formed by one-pot method. The sample was excited with UV light after complete evaporation of the solvent .

File Name: Supplementary Movie 4

Description: Real-time evolution process of **1-4** ill-defined nanorod heterostructures formed by one-pot method.
